# Supplementary material for: Hypertension as a sequela in patients of SARS-CoV-2 infection
Source: PLoS One. 2021 Apr 28;16(4):e0250815. doi: 10.1371/journal.pone.0250815 (PMC8081193; doi:10.1371/journal.pone.0250815)

PLOS ONE

Hypertension as a sequela in patients of SARS-CoV-2 infection

--Manuscript Draft--

Manuscript Number:	PONE-D-21-02635
Article Type:	Research Article
Full Title:	Hypertension as a sequela in patients of SARS-CoV-2 infection
Short Title:	SARS-CoV-2 with cardiac injury and hypertension
Corresponding Author:	Dan Hu<br>Wuhan University Renmin Hospital<br>Wuhan, CHINA
Keywords:	SARS-CoV-2; COVID-19; Cardiac injury; Hypertension; Risk factor
Abstract:	Background<br><br>COVID-19 is a respiratory infectious disease caused by SARS-CoV-2, and cardiovascular damage is commonly observed in affected patients. We sought to investigate the effect of SARS-CoV-2 infection on cardiac injury and hypertension during the current coronavirus pandemic.<br><br>Study design and methods<br><br>The clinical data of 366 hospitalized COVID-19-confirmed patients were analyzed. The clinical signs and laboratory findings were extracted from electronic medical records. Two independent, experienced clinicians reviewed and analyzed the data.<br><br>Results<br><br>Cardiac injury was found in 11.19% (30/268) of enrolled patients. 93.33% (28/30) of cardiac injury cases were in the severe group. The laboratory findings indicated that white blood cells, neutrophils, procalcitonin, C-reactive protein, lactate and lactic dehydrogenase were positively associated with cardiac injury marker. Of the 190 qualified patients, 16 (8.42%) patients without prior hypertension had a rise in blood pressure to the diagnostic criteria of hypertension during hospitalization, with a significantly increased level of the cTnI and procalcitonin. Eight of them (8/16, 50.00%) developed hypertension after four weeks of the follow-up visit. Multivariate analysis indicated that elevated age, cTnI, the presence of hypertension and diabetes were independent predictors for illness severity. The predictive model, based on the four parameters and gender, has a good ability to identify the clinical severity of COVID-19 in hospitalized patients (area under the curve: 0.932, sensitivity: 98.67%, specificity: 75.68%).<br><br>Conclusion<br><br>The cardiac injury of COVID-19 is likely to be related to secondary bacterial infection and hypoxia caused by respiratory failure. Hypertension, sometimes accompanied by elevated cTnI, may occur in patients of SARS-CoV-2 infection and become a sequela.
Order of Authors:	Ganxiao Chen
	Xun Li
	Zuojiong Gong
	Hao Xia
	Yao Wang
	Xuefen Wang
	Yan Huang
	Hector Barajas Martinez

Powered by Editorial Manager® and ProduXion Manager® from Aries Systems Corporation

	Dan Hu
**Additional Information:**	
**Question**	**Response**
**Financial Disclosure**<br><br>Enter a financial disclosure statement that describes the sources of funding for the work included in this submission. Review the submission guidelines for detailed requirements. View published research articles from *PLOS ONE* for specific examples.<br><br>This statement is required for submission and **will appear in the published article** if the submission is accepted. Please make sure it is accurate.<br><br>**Unfunded studies**<br>Enter: *The author(s) received no specific funding for this work.*<br><br>**Funded studies**<br>Enter a statement with the following details:<br>• Initials of the authors who received each award<br>• Grant numbers awarded to each author<br>• The full name of each funder<br>• URL of each funder website<br>• Did the sponsors or funders play any role in the study design, data collection and analysis, decision to publish, or preparation of the manuscript?<br>• **NO** - Include this sentence at the end of your statement: *The funders had no role in study design, data collection and analysis, decision to publish, or preparation of the manuscript.*<br>• **YES** - Specify the role(s) played.<br><br>* typeset	No - The funders had no role in study design, data collection and analysis, decision to publish, or preparation of the manuscript.
**Competing Interests**<br><br>Use the instructions below to enter a competing interest statement for this submission. On behalf of all authors, disclose any competing interests that could be perceived to bias this work—acknowledging all financial support and any other relevant financial or non-financial competing interests.	The authors report no relationships that could be construed as a conflict of interest.

Powered by Editorial Manager® and ProduXion Manager® from Aries Systems Corporation

<table>
<tr>
<td>This statement is required for submission and will appear in the published article if the submission is accepted. Please make sure it is accurate and that any funding sources listed in your Funding Information later in the submission form are also declared in your Financial Disclosure statement.

View published research articles from PLOS ONE for specific examples.

NO authors have competing interests

Enter: The authors have declared that no competing interests exist.

Authors with competing interests

Enter competing interest details beginning with this statement:

I have read the journal's policy and the authors of this manuscript have the following competing interests: [insert competing interests here]

* typeset</td>
<td></td>
</tr>
<tr>
<td>Ethics Statement

Enter an ethics statement for this submission. This statement is required if the study involved:

• Human participants
• Human specimens or tissue
• Vertebrate animals or cephalopods
• Vertebrate embryos or tissues
• Field research

Write "N/A" if the submission does not require an ethics statement.

General guidance is provided below. Consult the submission guidelines for detailed instructions. Make sure that all</td>
<td>This study was reviewed and approved by the Medical Ethical Committee of Renmin Hospital of Wuhan University.</td>
</tr>
</table>

Powered by Editorial Manager® and ProduXion Manager® from Aries Systems Corporation

information entered here is included in the Methods section of the manuscript.<br><br>**Format for specific study types**<br><br>**Human Subject Research (involving human participants and/or tissue)**<br>• Give the name of the institutional review board or ethics committee that approved the study<br>• Include the approval number and/or a statement indicating approval of this research<br>• Indicate the form of consent obtained (written/oral) or the reason that consent was not obtained (e.g. the data were analyzed anonymously)<br><br>**Animal Research (involving vertebrate animals, embryos or tissues)**<br>• Provide the name of the Institutional Animal Care and Use Committee (IACUC) or other relevant ethics board that reviewed the study protocol, and indicate whether they approved this research or granted a formal waiver of ethical approval<br>• Include an approval number if one was obtained<br>• If the study involved *non-human primates*, add *additional details* about animal welfare and steps taken to ameliorate suffering<br>• If anesthesia, euthanasia, or any kind of animal sacrifice is part of the study, include briefly which substances and/or methods were applied<br><br>**Field Research**<br><br>Include the following details if this study involves the collection of plant, animal, or other materials from a natural setting:<br>• Field permit number<br>• Name of the institution or relevant body that granted permission	
**Data Availability**<br><br>Authors are required to make all data underlying the findings described fully available, without restriction, and from the time of publication. PLOS allows rare exceptions to address legal and ethical	Yes - all data are fully available without restriction

concerns. See the PLOS Data Policy and FAQ for detailed information.<br><br>A Data Availability Statement describing where the data can be found is required at submission. Your answers to this question constitute the Data Availability Statement and **will be published in the article**, if accepted.<br><br>**Important:** Stating 'data available on request from the author' is not sufficient. If your data are only available upon request, select 'No' for the first question and explain your exceptional situation in the text box.<br><br>Do the authors confirm that all data underlying the findings described in their manuscript are fully available without restriction?	
**Describe where the data may be found in full sentences. If you are copying our sample text, replace any instances of XXX with the appropriate details.**<br><br>• If the data are **held or will be held in a public repository**, include URLs, accession numbers or DOIs. If this information will only be available after acceptance, indicate this by ticking the box below. For example: *All XXX files are available from the XXX database (accession number(s) XXX, XXX.*).<br>• If the data are all contained **within the manuscript and/or Supporting Information files**, enter the following: *All relevant data are within the manuscript and its Supporting Information files.*<br>• If neither of these applies but you are able to provide **details of access elsewhere**, with or without limitations, please do so. For example:<br><br>*Data cannot be shared publicly because of [XXX]. Data are available from the XXX Institutional Data Access / Ethics Committee (contact via XXX) for researchers who meet the criteria for access to confidential data.*	All datasets generated for this study are available from the corresponding author upon reasonable request.

Powered by Editorial Manager® and ProduXion Manager® from Aries Systems Corporation

The data underlying the results presented in the study are available from (include the name of the third party and contact information or URL).<br>• This text is appropriate if the data are owned by a third party and authors do not have permission to share the data.<br><br>* typeset	
Additional data availability information:	

Manuscript

Click here to access/download;Manuscript;Blood pressure in patients with COVID-19 - ver25.docx

Hypertension as a sequela in patients of SARS-CoV-2 infection

Short title：　Chen, et al. - SARS-CoV-2 with cardiac injury and hypertension

Ganxiao Chen, MD[1*], Xun Li, MD[2*], Zuojiong Gong, MD, PhD[2], Hao Xia, MD PhD [1], Yao Wang, MD [2], Xuefen Wang, MS [3], Yan Huang, MD, PhD [1], Hector Barajas-Martínez, PhD, FAHA, FHRS[4], Dan Hu, MD, PhD, FACC, FAHA, FHRS[1#]

1. Department of Cardiology & Cardiovascular Research Institute, Renmin Hospital of Wuhan University, Wuhan, 430060, China
2. Department of Infectious Diseases, Renmin Hospital of Wuhan University, Wuhan, 430060, China
3. Nursing department, Renmin Hospital of Wuhan University, Wuhan, 430060, China
4. Lankenau Institute for Medical Research, and Lankenau Heart Institute, Wynnewood, Pennsylvania and Jefferson Medical College, Philadelphia, Pennsylvania, USA

*These 2 authors contribute equally as first author.

[#]Corresponding author:
Dan Hu, MD, PhD, Professor,
FAHA, FACC, FHRS, FAPHRS
Associate Editor of *Frontiers in Physiology*
Department of Cardiology & Cardiovascular Research Institute,
Renmin Hospital of Wuhan University,
238 Jiefang Road, Wuhan, 430060, China.
Email: hudan0716@hotmail.com, or rm002646@whu.edu.cn
Phone: 86-27-88041911
Fax: 86-27-88042292

Word count: 3,126; **Abstract:** 258; **Figures:** 4; **Table:** 7; **References:** 30.

Abstract

Background: COVID-19 is a respiratory infectious disease caused by SARS-CoV-2, and cardiovascular damage is commonly observed in affected patients. We sought to investigate the effect of SARS-CoV-2 infection on cardiac injury and hypertension during the current coronavirus pandemic.

Study design and methods: The clinical data of 366 hospitalized COVID-19-confirmed patients were analyzed. The clinical signs and laboratory findings were extracted from electronic medical records. Two independent, experienced clinicians reviewed and analyzed the data.

Results: Cardiac injury was found in 11.19 % (30/268) of enrolled patients. 93.33% (28/30) of cardiac injury cases were in the severe group. The laboratory findings indicated that white blood cells, neutrophils, procalcitonin, C-reactive protein, lactate and lactic dehydrogenase were positively associated with cardiac injury marker. Of the 190 qualified patients, 16 (8.42%) patients without prior hypertension had a rise in blood pressure to the diagnostic criteria of hypertension during hospitalization, with a significantly increased level of the cTnI and procalcitonin. Eight of them (8/16, 50.00%) developed hypertension after four weeks of the follow-up visit. Multivariate analysis indicated that elevated age, cTnI, the presence of hypertension and diabetes were independent predictors for illness severity. The predictive model, based on the four parameters and gender, has a good ability to identify the clinical severity of COVID-19 in hospitalized patients (area under the curve: 0.932, sensitivity: 98.67%, specificity: 75.68%).

Conclusion: The cardiac injury of COVID-19 is likely to be related to secondary bacterial infection and hypoxia caused by respiratory failure. Hypertension, sometimes accompanied by elevated cTnI, may occur in patients of SARS-CoV-2 infection and become a sequela.

Keywords: SARS-CoV-2; COVID-19; Cardiac injury; Hypertension; Risk factor

Introduction

In December 2019, an acute respiratory infectious disease known as "coronavirus disease 2019 (COVID-19)" caused by a novel coronavirus occurred in Wuhan, China [1,2]. Whole-genome sequencing and systematic analysis showed that this novel. Coronavirus is a distinct clade from beta coronavirus associated with human severe acute respiratory syndrome (SARS) and Middle East respiratory syndrome (MERS)[3], and was now officially named "SARS-CoV-2" by World Health Organization. Both SARS-CoV and SARS-CoV-2 have been identified to use angiotensin converting enzyme II (ACE2) receptor as the portal of entry into the affected cell[4,5]. ACE2, a membrane-bound aminopeptidase, is highly expressed in the heart and lung[6,7]. Although the main clinical features of COVID-19 are dominated by respiratory symptoms, many patients with severe cardiovascular damage have been reported by our team and others[8,9]. Besides, patients with underlying cardiovascular diseases (CVDs) might have an increased risk of death[8,10]. So, understanding the damage to the cardiovascular system caused by SARS-CoV-2 and the underlying mechanisms is of great importance so that these patients can be treated timely, and the mortality can be reduced. In this retrospective cohort study, the clinical data of hospitalized COVID-19-confirmed patients were analyzed to explore the consequences of SARS-CoV-2 infection on the cardiovascular system.

Materials and methods

Study setting and population

There were 366 COVID-19-confirmed patients enrolled in this study, who hospitalized in the Department of Infectious Diseases, Renmin Hospital of Wuhan University, from February 1 to May 1, 2020. Clinical severity was defined for all enrolled COVID-19 patients according to the guidelines of the National Health Commission of China, including four types as mild, moderate, severe, and critical types[11]. We divided the patients into the non-severe group (mild and moderate types) and the severe group (severe and critical type). Patients with one of the three conditions ~~are~~ considered as severe type: respiratory distress and respiratory rate higher than 30 times per minute; fingertip blood oxygen saturation less than 93% at rest; partial arterial oxygen pressure (PaO2) / fraction of inspiration oxygen (FiO2) less than 300mmHg. Patients in critical type ~~should meet~~ one of the following criteria: respiratory failure, requiring mechanical ventilation; shock; multiple organ failure, requiring intensive care management. This study was reviewed and approved by the Medical Ethical Committee of Renmin Hospital of Wuhan University.

Data collection

The clinical signs and laboratory findings were extracted from electronic medical records. Two independent, experienced clinicians reviewed and abstracted the data. The recorded information ~~includes~~ demographic data, potential comorbidities, symptoms, signs, laboratory test results. The serum level of hypersensitive troponin I (cTnI) exceeding >40 pg/mL was considered cardiac injury. Blood pressures were obtained three fixed times in the morning using standard measurement. Hypertension was defined as previous history of hypertension, use of antihypertensive drugs, or

brachial blood pressure ≥ 140 mm / 90 mmHg. For patients without prior hypertension, elevated blood pressure was defined as blood pressure ≥ 140 mm / 90 mmHg for more than 3 times during hospitalization.

The processes of patient screening

The screening process for evaluating the effect of SARS-CoV-2 on the cardiovascular system is shown in **Figure 1**. Serum level of cardiac troponin I (cTnI) was tested in 276 of the 366 patients during hospitalization, among which 8 patients had a history of chronic heart disease. Thus, 268 patients were enrolled to evaluate the effect of SARS-CoV-2 on cardiac injury. Of the 366 patients, 278 had complete blood pressure data. Among which, 88 patients had a history of hypertension before hospitalization; therefore, 190 patients were grouped to evaluate the effect of SARS-CoV-2 on blood pressure. Among all 366 subjects, 194 subjects had data available on serum level of cTnI and complete blood pressure data. After the exclusion of the case with a history of chronic heart disease, 186 cases were included to evaluate the association between cTnI, blood pressure, and clinical severity of COVID-19.

Statistical analysis

Student's *t-test* or the Mann-Whitney test was used to compare the mean of continuous variables, *Fisher's* exact test was used with limited data, the $\chi 2$ test was used to compare the proportion of categorical variables. Spearman correlation analysis was used to analyze the correlation between variables. The logistic regression model was used to determine factors associated with the clinical severity of COVID-19, and the analysis of receiver operating characteristic (ROC) curves were constructed

according to standard procedures. The Youden index, defined as (sensitivity + specificity) – 1, was used to derive a reasonable cut-off value. Calibration of the risk prediction model, comparing the observed and predicted probability, was performed via a visual calibration plot in the R program. A P-value of <0.05 was considered statistically significant. Statistical analysis was carried out using SPSS software version 21.0 and R version 3.0.

Results

The effect of sars-cov-2 on cardiac injury

The results showed that the median age of patients with or without cardiac injury was 74 y/o and 49 y/o with statistical significance. The male was dominant in the cardiac injury group (86.67 %). The cardiac injury was found in 11.19 % (30/268) of patients, but 93.33 % (28/30) of them were in the severe group. Only 1.75 % (2/114) of patients in the non-severe group suffered from cardiac injury. Moreover, 66.67 % (20/30) of cardiac injury patients in the severe group eventually died. The most frequent symptom of patients was fever, followed by cough, fatigue, dyspnea, and chest stuffiness. The incidences of cough, dyspnea, and chest stuffiness were significantly different between the patients with or without cardiac injury ($P < 0.05$). Hypertension was the most frequent comorbidity, while the incidence of diabetes and the malignant tumor was significantly different between the two groups ($P < 0.05$, **Table. 1**). The laboratory findings indicated that the patients who suffered from cardiac injury had a higher level of white blood cells, neutrophils, monocytes, procalcitonin, C-reactive

protein, lactate, and lactic dehydrogenase compared with the patients without cardiac injury (P < 0.05). Correlation analysis showed that white blood cells, neutrophils procalcitonin, C-reactive protein, lactate and lactic dehydrogenase were significantly associated with cTnI (P < 0.05), the r values were 0.515 [95% CI, 0.394 - 0.632], 0.486 [95% CI, 0.358 - 0.591], 0.477 [95% CI, 0.352 - 0.581], 0.459 [95% CI, 0.338 - 0.566], 0.424 [95% CI, 0.273 - 0.559] and 0.438 [95% CI, 0.291 - 0.561], respectively (**Table 2**).

The effect of SARS-CoV-2 on blood pressure

Of the 190 qualified patients, 16 (8.42%) patients had a rise in blood pressure during hospitalization, among which 6 patients were male, and 10 patients were female. As shown in **Table 3**, no significant differences were found when comparing the ~~basic information~~, including age, gender, clinical category, symptoms, and comorbidities between patients with or without elevated blood pressure (P > 0.05). Compared with the patients without elevated blood pressure, the level of cTnI and procalcitonin in the 16 patients rose significantly (P < 0.05, **Table 4)**. Elevated systolic blood pressure was observed in most of the patients, while diastolic blood pressure was in the normal range. The median values of blood pressure and plasma cTnI levels changes of the 16 patients are shown in **Figure 2A**. Systolic blood pressure and cTnI levels had a similar trend with the treatment time. In addition, the blood pressure, cTnI, and white blood cells were continuously monitored in one index case (**Figure 2B**). With effective treatment, the patient's condition improved ~~according to symptoms and chest computed tomography~~ (CT). Meanwhile, the

systolic blood pressure and white blood cells reverted to the normal range, and the concentrations of cTnI were also gradually reduced. After discharge from the hospital, 8 patients got recovery after 4 weeks of follow-up visits, and the other 8 patients developed hypertension. The median ages of patients who recovered from or got hypertension were 45.50 y/o and 66.50 y/o, respectively. The difference in the age between these two groups was statistically significant (*P* < 0.05). Among those who got hypertension, 6 patients took antihypertensive drugs, 4 of whom were treated with the antihypertensive drugs before discharge from the hospital, and 2 of whom began to take bisoprolol one week after discharge. Among the 190 patients without prior hypertension, 30 patients detected the serum levels of adrenocorticotrophic hormone, renin, angiotensin Ⅱ (Ang Ⅱ), and aldosterone. As shown in **Table 5**, the serum renin levels of all tested patients were in the normal range, while 73.33% (22/30) of the patients had an elevated Ang Ⅱ level. Among those 22, 6 (27.27%) patients had a rise in blood pressure.

The association between cardiac Injury, blood pressure, and clinical severity of COVID-19.

The comparison of clinical and laboratory findings was completed between the severe and non-severe group (**Table 6**). Patients in the severe group were significantly older, with a greater proportion of males (P < 0.05). Besides, the cTnI, white blood cells, neutrophils, procalcitonin, C-reactive protein, and lactic dehydrogenase of the severe group were significantly higher than those of the non-severe group. In contrast, patients in the severe group had a significantly lower level of lymphocytes. Further

univariate analysis revealed that the age, sex, cTnI, white blood cells, neutrophils, lymphocytes, C-reactive protein, lactic dehydrogenase, and history of hypertension and diabetes were associated with the clinical severity of COVID-19. In the multivariate analysis, the age, cTnI and history of hypertension and diabetes remained significant independent predictors (OR=1.11, 95% CI: 1.07 - 1.16, P < 0.001; OR = 1.08, 95% CI: 1.01 - 1.15, P = 0.018; OR = 7.19, 95% CI: 2.55 - 20.31, P < 0.001; OR=4.28, 95% CI: 1.41 - 12.97, P = 0.010; **Table 7**). The receiver operating characteristic curve of the four independent predictors and gender for clinical severity of COVID-19 is shown in **Figures 3A** (AUC: 0.932, sensitivity: 98.67 %, specificity: 75.68 %). The calibration indicated that the model was well-calibrated (**Figure 3B**).

Discussion

The SARS-CoV-2 has been identified as one of a class of single-stranded enveloped 39 RNA viruses, which belongs to the beta-coronaviruses genus of the coronaviridae family[3]. These coronaviruses have a three-dimensional structure of spike protein, which is closely bound to human cell ACE2 receptor. Therefore, the cells with ACE2 expression may act as target cells and be susceptible to SARS-CoV-2 infection[12]. ACE2 is a membrane-bound aminopeptidase that has a vital role in cardiovascular system[13, 14]. It is reasonable to speculate that the SARS-CoV-2 will act on the heart and blood vessels, resulting in subsequent changes in the cardiovascular system.

In this retrospective cohort study, the assay of blood levels of cardiac troponins is the recommended procedure for the detection of cardiac injury in patients[15]. Huang and

colleagues find that cTnI is increased substantially in 12.20 % (5/41) Wuhan COVID-19 patients, in whom the diagnosis of the virus-related cardiac injury is made[8]. Another previous research also reported that patients with cardiac injury had higher levels of leukocyte counts, C-reactive protein, procalcitonin[16]. In this study, older patients with diabetes and malignant tumor are more likely to suffer from cardiac injury. Our further analysis shows that the level of white blood cells, neutrophils, procalcitonin, C-reactive protein, lactate, and lactic dehydrogenase were positively associated with cardiac injury, which indicates that the cardiac injury of COVID-19 patients might be related to secondary bacterial infection and hypoxia caused by respiratory failure. In COVID-19, potential reasons for cTnI increase are mainly concentrated on chronic myocardial injury, acute nonischemic myocardial injury, and acute myocardial infarction[17]. Although a case report has suggested that cardiac involvement is a complication associated with COVID-19[18]. For reasons known to all, our understanding of pathogenesis and complications of COVID-19 is limited at present. As concerning the effect of SARS-CoV-2 on the cardiovascular system, it is not clear whether heart damage is related to viral load due to the lack of virus quantification results. Besides reporting that cTnI values above the 99th percentile of the reference upper limit were observed in approximately 10% of positive cases, a meta-analysis has shown the values of cTnI are significantly increased in COVID-19 patients with severe disease[19]. This is similar to our results, but we furtherly highlight that the level of cardiac injury was independent predictors and combine the age and comorbidities to construct a logistic regression model to identify the clinical severity of COVID-19 in hospitalized patients.

Sixteen patients without prior hypertension ~~have~~ a rise in blood pressure during hospitalization, and higher systolic blood pressure ~~is~~ observed in most of the patients. Except for lymphocytes and procalcitonin, no significant differences are found in patients with and without elevated blood pressure. ~~It~~ suggests that abnormal blood pressure ~~might not only be caused by inflammatory responses.~~ ~~The~~ laboratory results of renin-angiotensin system (RAS) ~~show~~ that Ang II levels are elevated in the majority of patients without prior hypertension (73.33%), while the serum renin levels of all the patients ~~are~~ in the normal range. The results indicate that the rise of blood pressure ~~may associate with the elevated Ang II levels.~~ The RAS plays a critical role in the cardiovascular system, which includes a classical RAS axis (ACE-Ang II-AT1R pathway) and a non-classical RAS axis (ACE2-Ang 1-7-MasR-based pathway), counter-balancing role of the two axes regulates cardiovascular physiology and disease[20, 21]. ACE2 cleaves Ang II into the Ang 1-7, thus limiting substrate availability in the adverse ACE/Ang II/AT1 receptor axis[22, 23]. Keidar and colleagues ~~find~~ that ~~the~~ ACE2 ~~protects against hypertension is probably achieved by the degradation of~~ Ang II[24]. In the present study, the rise in blood pressure of ~~the~~ COVID-19 patients may be associated with SARS-CoV-2 infection, since ACE2 is a receptor of SARS-CoV-2. ~~The mechanism may be that SARS-CoV-2 binds and inhibits ACE2,~~ inhibiting the degradation of Ang II, thereby causing vasoconstriction and elevated blood pressure. Another hypothesis is that over activation of the RAS system promotes inflammatory response and cytokine storm, which stimulates the NADH/NADPH oxidase system and triggers cell contraction and vasoconstriction, ~~then~~ leads to COVID-19 related lung

injury. Though the underlying mechanism remains to be elucidated, ~~it's sure the~~ RAS plays a major role in hypertension and COVID-19 infection, ~~which is what we observe in our study.~~ It has been noticed that recombinant human ACE2 is considered ~~as~~ a treatment for patients with COVID-19 (*ClinicalTrials.gov ID: NCT04287686*). This finding probably shades important implications for future treatment strategies. A recent long-term observational follow-up study of patients with COVID-19 reported nearly one eighth ~~without renal dysfunction~~ ~~had a~~ reduction in glomerular filtration rate at follow-up. In addition, COVID-19 survivors suffer from relatively higher levels of depressive, anxious, and somatic symptoms (including fatigue or muscle weakness). Severe cases are more susceptible to ~~develop~~ reduced pulmonary diffusion capacities[25]. Multiple above factors are capable of inducing hypertension in nonhypertensive ~~ones~~. In addition, the median ages of these patients ~~are~~ 66.5 y/o. It seems that SARS-CoV-2 infection is just a trigger, and age plays a more important role.

On the other hand, sixteen patients with elevated blood pressure show higher levels of cTnI, which indicates that elevated blood pressure could occur in parallel with mild cardiac injury of COVID-19 patients. Concerning the link between cTnI and hypertension, most recent studies have focused on patients with decreased left ventricular ejection fraction or increased left ventricular mass index[26]. Serum heart-type fatty acid-binding protein levels, a sensitive marker of myocardial damage, is found to be significantly implicated in arterial stiffness in newly diagnosed hypertensive individuals[27]. Likewise, in spontaneously hypertensive rats, angiotensin II acting on its type 1/2 receptors is known to participate in myocardial injury by

mediating post-transcriptional processing and the subsequent accumulation of proapoptotic protein Bax-α[28]. Thus, we reasonably deduct that elevated Ang II levels in COVID-19 patients, especially in newly diagnosed hypertensive ones, could lead to the up-regulation of the pathway, which causes cardiac impairment.

Study strength and limitations

Hypertension, as the most common comorbidities, is an independent risk factor associated with COVID-19 patients[29,30], but the effect of SARS-CoV-2 on hypertension has rarely been reported. In the present study, we propose that hypertension is probably a sequela of SARS-CoV-2 infection. Although a number of studies of COVID-19 have been reported, there are few reports about the sequela of the disease by far due to lack of long-term clinical follow-up, which also applies to our present research. Next, many patients could not be incorporated in the analysis because of history of hypertension, which results in a relatively low number. Whereas, our emphasis is that spontaneous hypertension becomes a consequence of COVID-19 and accompanies with even higher cTnI level, which is definitely rare and different from previous statement of hypertension in general COVID-19 subjects.

Conclusion

In summary, SARS-CoV-2 may impair the cardiomyocytes in certain pathways; the cardiac injury of COVID-19 is likely to be related to secondary bacterial infection and hypoxia caused by respiratory failure. As a clinical index reflecting damage to cardiac injury, cTnI is correlated with severity of the infection. As presented in **Striking image**, Spontaneous hypertension may occur in patients, accompanied by

mild elevation in cTnI, during hospitalization and could become a sequela of SARS-CoV-2 infection, which may warrant more aggressive monitoring and management of blood pressure.

Acknowledgment: We gratefully acknowledge contributions from all enrolled patients and related medical staff.

Data Availability Statement: All datasets generated for this study are available from the corresponding author upon reasonable request.

Funding: The current work was supported by the National Natural Science Foundation Project of China (Grant No. 81670304 – D.H.).

Competing interests: The authors report no relationships that could be construed as a conflict of interest.

Abbreviations: ACE2, angiotensin converting enzyme II; Ang II, Angiotensin II; AT1R, Angiotensin II type-1 receptor; COVID-19, coronavirus disease 2019; cTnI, cardiac troponin I; CVDs, cardiovascular diseases; MERS, middle east respiratory syndrome; RAS, renin-angiotensin system; SARS, severe acute respiratory syndrome.

Authors Contributions

Conceptualization: Ganxiao Chen, Xun Li, Zuojiong Gong, Hao Xia, Yao Wang, Xuefen Wang, Yan Huang, Hector Barajas-Martínez, Dan Hu.

Data curation: Ganxiao Chen, Xun Li, Yao Wang, Xuefen Wang.

Formal analysis: Ganxiao Chen, Xun Li.

Investigation: Ganxiao Chen, Xun Li, Zuojiong Gong.

Methodology: Zuojiong Gong, Hao Xia, Yan Huang, Hector Barajas-Martínez, Dan Hu.

Project administration: Dan Hu.

Software: Ganxiao Chen, Xun Li.

Supervision: Dan Hu.

Validation: Dan Hu.

Visualization: Ganxiao Chen, Xun Li.

Writing - original draft: Ganxiao Chen, Xun Li, Zuojiong Gong, Hao Xia.

Writing - review & editing: Ganxiao Chen, Xun Li, Zuojiong Gong, Hao Xia, Yao Wang, Xuefen Wang, Yan Huang, Hector Barajas-Martínez, Dan Hu.

.

References

1. Lu H, Stratton CW, and Tang YW. Outbreak of pneumonia of unknown etiology in Wuhan, China: The mystery and the miracle. *J Med Virol*. (2020) 92:401-402. doi:10.1002/jmv.25678

2. Hui DS, E IA, Madani TA, Ntoumi F, Kock R, Dar O, et al. The continuing 2019-nCoV epidemic threat of novel coronaviruses to global health - The latest 2019 novel coronavirus outbreak in Wuhan, China. *Int J Infect Dis*. (2020) 91:264-266. doi:10.1016/j.ijid.2020.01.009

3. Zhu N, Zhang D, Wang W, Li X, Yang B, Song J, et al. A Novel Coronavirus from Patients with Pneumonia in China, 2019. *N Engl J Med*. (2020) 382:727-733. doi:10.1056/NEJMoa2001017

4. Brojakowska A, Narula J, Shimony R and Bander J. Clinical Implications of SARS-CoV-2 Interaction With Renin Angiotensin System: JACC Review Topic of the Week. *J Am Coll Cardiol*. (2020) 75:3085-3095. doi:10.1016/j.jacc.2020.04.028

5. Chen Y, Guo Y, Pan Y and Zhao ZJ. Structure analysis of the receptor binding of 2019-nCoV. *Biochem Biophys Res Commun*. (2020) 525(1):135-140. doi:10.1016/j.bbrc.2020.02.071

6. Gheblawi M, Wang K, Viveiros A, Nguyen Q, Zhong JC, Turner AJ, et al. Angiotensin-Converting Enzyme 2: SARS-CoV-2 Receptor and Regulator of the Renin-Angiotensin System: Celebrating the 20th Anniversary of the Discovery of ACE2. *Circ Res*. (2020) 126:1456-1474. doi:10.1161/CIRCRESAHA.120.317015

7. Keidar S, Kaplan M and Gamliel-Lazarovich A. ACE2 of the heart: From

angiotensin I to angiotensin (1-7). *Cardiovasc Res*. (2007) 73:463-9. doi:10.1016/j.cardiores.2006.09.006

8. Huang C, Wang Y, Li X, Ren L, Zhao J, Hu Y, et al. Clinical features of patients infected with 2019 novel coronavirus in Wuhan, China. *Lancet*. (2020) 395:497-506. doi:10.1016/S0140-6736(20)30183-5

9. Hu D, Liu K, Li BX, Hu ZH. Large Intra-cardiac Thrombus in a COVID-19 Patient Treated with Prolonged Extracorporeal Membrane Oxygenation Implantation. Eur Heart J. (2020) 41(32):3104-3105. doi:10.1093/eurheartj/ehaa524.

10. Chen T, Wu D, Chen H, Yan W, Yang D, Chen G, et al. Clinical characteristics of 113 deceased patients with coronavirus disease 2019: retrospective study. *BMJ*. (2020) 368:m1091. doi:10.1136/bmj.m1091

11. National Health and Health Commission and State Administration of traditional Chinese medicine. Diagnosis and treatment of pneumonia caused by new coronavirus infection(Trial version 5[J/OL]). *Chinese J Integrated Traditional Western Edicine*. (2020) 40:136–8. doi:10.7661/j.cjim.20200202.064

12. Zhou P, Yang XL, Wang XG, Hu B, Zhang L, Zhang W, et al. A pneumonia outbreak associated with a new coronavirus of probable bat origin. *Nature*. (2020) 579:270-273. doi:10.1038/s41586-020-2012-7

13. Turner AJ, Hiscox JA and Hooper NM. ACE2: from vasopeptidase to SARS virus receptor. *Trends Pharmacol Sci*. (2004) 25:291-4. doi:10.1016/j.tips.2004.04.001

14. Hamming I, Cooper ME, Haagmans BL, Hooper NM, Korstanje R, Osterhaus AD, et al. The emerging role of ACE2 in physiology and disease. *J Pathol*. (2007) 212:1-

11. doi:10.1002/path.2162

15. Venge P, Johnston N, Lindahl B and James S. Normal plasma levels of cardiac troponin I measured by the high-sensitivity cardiac troponin I access prototype assay and the impact on the diagnosis of myocardial ischemia. *J Am Coll Cardiol*. (2009) 54:1165-72. doi:10.1016/j.jacc.2009.05.051

16. Shi S, Qin M, Shen B, Cai Y, Liu T, Yang F, et al. Association of Cardiac Injury With Mortality in Hospitalized Patients With COVID-19 in Wuhan, China. *JAMA Cardiol*. (2020) 5:802-810. doi:10.1001/jamacardio.2020.0950

17. Sandoval Y, Januzzi JL, Jr. and Jaffe AS. Cardiac Troponin for Assessment of Myocardial Injury in COVID-19: JACC Review Topic of the Week. J Am Coll Cardiol. (2020) 76:1244-1258. doi:10.1016/j.jacc.2020.06.068

18. Inciardi RM, Lupi L, Zaccone G, Italia L, Raffo M, Tomasoni D, et al. Cardiac Involvement in a Patient With Coronavirus Disease 2019 (COVID-19). *JAMA Cardiol*. (2020) 5:819-824. doi:10.1001/jamacardio.2020.1096

19. Lippi G, Lavie CJ and Sanchis-Gomar F. Cardiac troponin I in patients with coronavirus disease 2019 (COVID-19): Evidence from a meta-analysis. *Prog Cardiovasc Dis*. (2020) 63:390-391. doi:10.1016/j.pcad.2020.03.001

20. Paz Ocaranza M, Riquelme JA, Garcia L, Jalil JE, Chiong M, Santos RAS, et al. Counter-regulatory renin-angiotensin system in cardiovascular disease. *Nat Rev Cardiol*. (2020) 17:116-129. doi:10.1038/s41569-019-0244-8

21. Te Riet L, van Esch JH, Roks AJ, van den Meiracker AH and Danser AH. Hypertension: renin-angiotensin-aldosterone system alterations. *Circ Res*. (2015)

116:960-75. doi:10.1161/CIRCRESAHA.116.303587

22. Wang K, Gheblawi M and Oudit GY. Angiotensin Converting Enzyme 2: A Double-Edged Sword. *Circulation*. (2020) 142:426–428. doi:10.1161/CIRCULATIONAHA.120.047049

23. Chappell MC, Marshall AC, Alzayadneh EM, Shaltout HA and Diz DI. Update on the Angiotensin converting enzyme 2-Angiotensin (1-7)-MAS receptor axis: fetal programing, sex differences, and intracellular pathways. *Front Endocrinol (Lausanne)*. (2014) 4:201. doi:10.3389/fendo.2013.00201

24. Keidar S, Strizevsky A, Raz A and Gamliel-Lazarovich A. ACE2 activity is increased in monocyte-derived macrophages from prehypertensive subjects. *Nephrol Dial Transplant*. (2007) 22:597-601. doi:10.1093/ndt/gfl632

25. Huang C, Huang L, Wang Y, Li X, Ren L, Gu X, et al. 6-month consequences of COVID-19 in patients discharged from hospital: a cohort study. *Lancet*. (2021) 397:220-232. doi:10.1016/S0140-6736(20)32656-8

26. Hamwi SM, Sharma AK, Weissman NJ, Goldstein SA, Apple S, Canos DA, et al. Troponin-I elevation in patients with increased left ventricular mass. Am J Cardiol. (2003) 92:88-90. doi:10.1016/s0002-9149(03)00477-6

27. Gedikli O, Ozturk S, Yilmaz H, Baykan M, Kiris A, Durmus I, et al. Relationship between arterial stiffness and myocardial damage in patients with newly diagnosed essential hypertension. Am J Hypertens. (2008) 21:989-93. doi:10.1038/ajh.2008.235

28. Ravassa S, Fortuno MA, Gonzalez A, Lopez B, Zalba G, Fortuno A, et al. Mechanisms of increased susceptibility to angiotensin II-induced apoptosis in

ventricular cardiomyocytes of spontaneously hypertensive rats. Hypertension. (2000) 36:1065-71. doi:10.1161/01.hyp.36.6.1065

29. Wang B, Li R, Lu Z and Huang Y. Does comorbidity increase the risk of patients with COVID-19: evidence from meta-analysis. *Aging (Albany NY)*. (2020) 12:6049-6057. doi:10.18632/aging.103000

30. Karagiannidis C, Mostert C, Hentschker C, Voshaar T, Malzahn J, Schillinger G, et al. Case characteristics, resource use, and outcomes of 10 021 patients with COVID-19 admitted to 920 German hospitals: an observational study. Lancet Respir Med. (2020) 8:853-862. doi:10.1016/S2213-2600(20)30316-7

Figure legends

Figure 1. The flow diagram of patient screening.

Figure 2. A. The systolic blood pressure and cTnI change of patients with elevated blood pressure. B. The systolic blood pressure, cTnI, white blood cells, and chest computed tomography changes of one patient with elevated blood pressure. Late follow-up: The 4th week after discharge from the hospital.

Figure 3. A. ROC curves of the age, cTnI, gender, and the presence of hypertension and diabetes for the identification of the severity of COVID-19. B. The calibration plot for the comparison of the predicted and actual probability. The X-axis and Y-axis represent the model-predicted and actual probability of MAE, respectively. The red line: perfect prediction. The black line: predictive performance of the model after bootstrapping (B=1000 repetitions).

Striking image. The main findings of the present research.

Table 1. Clinical characteristics of COVID-19 patients with or without cardiac injury

	Total (n=268)	Cardiac injury	
		**Non-injury (n=238)**	**Injury (n=30)**
**Age (y/o), Median (IQR)**	53 (42 - 69)	49 (40 - 66)	74 (73 - 86)*
**Gender (n, %)**			
Male	144 (53.7)	118 (49.58)	26 (86.67)*
Female	124 (46.3)	120 (50.42)	4 (13.33)*
**Clinical categories (n, %)**			
Non-severe	114 (42.54)	112 (47.06)	2 (6.67)*
Severe	154 (57.46)	126 (52.94)	28 (93.33)*
**Symptoms (n, %)**			
Fever	224 (83.58)	196 (82.35)	28 (93.33)
Cough	192 (71.64)	164 (68.91)	28 (93.33)*
Dyspnea	120 (44.78)	92 (38.66)	28 (93.33)*
Chest stuffiness	114 (42.54)	88 (36.97)	26 (86.67)*
Fatigue	148 (55.22)	130 (54.62)	18 (60.00)
Muscle soreness	54 (20.15)	48 (20.17)	6 (20.00)
**Comorbidities (n, %)**			
Hypertension	94 (35.07)	80 (33.61)	14 (46.67)
Diabetes	44 (16.42)	34 (14.29)	10 (33.33)*
Chronic lung diseases	10 (3.73)	8 (3.36)	2 (6.67)
Chronic kidney diseases	4 (1.49)	2 (0.84)	2 (6.67)
Gastrointestinal diseases	2 (0.75)	2 (0.84)	0 (0)
Malignant tumor	4(1.49)	0 (0)	4 (13.33)*

Student's *t-test*, *χ2 test,* and *Fisher's exact tests* were used to compare the age, gender, clinical category, symptoms, and comorbidities between 2 groups. *P <0.05 is considered statistically significant.

Table 2. The laboratory findings of COVID-19 patients with or without cardiac injury.

Laboratory findings	Non-cardiac injury (median, IQR)	Cardiac injury (median, IQR)	Normal range	*r* values	*P* values
CTnI (pg/mL)	7.5 (6.00 - 16.50)	162 (68.20 - 757.50)*	0 - 40	1.000	< 0.001
White blood cells (×10^9, cells/L)	5.93 (4.45 - 7.06)	9.67 (5.62 - 13.73)*	3.5 - 9.5	0.515	< 0.001
Neutrophils (×10^9, cells/L)	3.72 (2.95 - 5.47)	5.52 (3.83 - 11.62)*	1.8 - 6.3	0.486	< 0.001
Lymphocytes (×10^9, cells/L)	1.01 (0.59 - 1.25)	0.67 (0.48 - 1.39)*	1.1 - 3.2	-0.230	0.001
Monocytes (×10^9, cells/L)	0.44 (0.25 - 0.69)	0.62 (0.42 - 0.76)*	0.1 - 0.6	0.127	0.080
Procalcitonin (pg/mL)	60 (32.00 - 121.00)	630 (47.00 - 2750.00)*	0 - 100	0.477	< 0.001
C-reactive protein (mg/L)	41.40 (5.00 - 74.40)	81.10 (14.20 - 142.80)*	0 - 10	0.459	< 0.001
Lactate (mmol/L)	1.70 (1.15 - 2.00)	2.10 (1.95 - 3.05)*	0.5 - 1.5	0.424	< 0.001
Lactic dehydrogenase (U/L)	275 (218.00 - 375.00)	428 (325.00 - 765.00)*	120 - 250	0.438	< 0.001

Student's *t* test or the Mann-Whitney test was used to compare the differences between non-cardiac injury and cardiac injury groups, *$P < 0.05$ is considered statistically significant. Spearman correlation analysis was used to analyze the correlation between the cTnI and other laboratory findings.

Table 3. Clinical characteristics of COVID-19 patients without prior hypertension

	Total (n=190)	Blood pressure	
		Normal (n=174)	Elevated (n=16)
**Age (y/o), Median (IQR)**	54 (39 - 63)	53 (39 - 63)	60 (42 - 70)
**Gender (n, %)**			
Male	78 (41.05)	72 (41.38)	6 (37.50)
Female	112 (58.95)	102 (58.62)	10 (62.50)
**Clinical categories (n, %)**			
Non-severe	116 (61.05)	108 (62.07)	8 (50.00)
Severe	74 (38.95)	66 (37.93)	8 (50.00)
**Symptoms (n, %)**			
Fever	156 (82.11)	142 (81.61)	14 (87.50)
Cough	132 (69.47)	120 (68.97)	12 (75.00)
Dyspnea	70 (36.84)	64 (36.78)	6 (37.50)
Chest stuffiness	68 (35.79)	62 (35.63)	6 (37.50)
Fatigue	96 (50.53)	88 (50.57)	8 (50.00)
Muscle soreness	36 (18.95)	34 (19.54)	2 (12.50)
**Comorbidities (n, %)**			
Diabetes	28 (14.74)	26 (14.94)	2 (12.50)
Chronic lung diseases	6 (3.16)	6 (3.45)	0 (0)
Gastrointestinal diseases	4 (2.11)	4 (2.30)	0 (0)
Thyroid disease	2 (1.05)	2 (1.15)	0 (0)
Prostate disease	2 (1.05)	2 (1.15)	0 (0)

Student's *t-test*, *χ2 test,* and *Fisher's exact tests* were used to compare the age, gender, clinical category, symptoms, and comorbidities between 2 groups. No significant differences were found.

Table 4. The laboratory findings of patients with or without elevated blood pressure

Laboratory findings	Normal blood pressure (median, IQR)	Elevated blood pressure (median, IQR)	Normal range
CTnI (pg/mL)	3.86 (2.49 - 5.15)	22.00 (18.20 - 30.00)*	0 - 40.00
White blood cells ($\times 10^9$, cells/L)	5.24 (3.87 - 7.00)	4.86 (3.96 - 6.60)	3.50 - 9.50
Neutrophils ($\times 10^9$, cells/L)	3.14 (2.48 - 4.96)	3.64 (3.06 - 4.94)	1.80 - 6.30
Lymphocytes ($\times 10^9$, cells/L)	1.13 (0.76 - 1.56)	0.93 (0.57 - 1.23)*	1.10 - 3.20
Monocytes ($\times 10^9$, cells/L)	0.44 (0.33 - 0.56)	0.55 (0.25 - 0.61)	0.10 - 0.60
Hemoglobin (g/L)	128 (118 - 136)	118(107 - 137)	115 - 150
Procalcitonin (pg/mL)	49 (28 - 73)	82 (53 - 430)*	0 - 100
C-reactive protein (mg/L)	20.00 (2.40 - 44.50)	7.80 (3.51 - 33.20)	0 - 10.00
Lactic dehydrogenase (U/L)	259 (206 - 313)	259 (208 - 289)	120 - 250

Student's *t* test or the Mann-Whitney test was used to compare the differences between non-hypertension and hypertension groups, *P <0.05 is considered statistically significant.

Table 5. The effect of SARS-CoV-2 infection on renin-angiotensin system in subjects without prior hypertension

Laboratory findings	Test results (median, IQR)	Normal range	Below the lower limit (n, %)	Above the upper limit (n, %)
Adrenocorticotrophic hormone (pg/mL)	27.96 (14.11 - 55.98)	7.20 - 63.40	2 (6.67)	2 (6.67)
Renin (pg/mL)	5.21 (3.60 - 8.39)	4.00 - 24.00	0 (0)	0 (0)
Angiotensin Ⅱ (pg/mL)	150.00 (125.10 - 170.90)	25.00 - 129.00	0 (0)	22 (73.33)
Aldosterone (pg/mL)	142.70 (118.50 - 163.80)	10.00 - 160.00	0 (0)	4 (13.33)

Note: n=30 in total subjects.

Table 6. Clinical characteristics of severe and non-severe COVID-19 patients

	Clinical Severity		Normal range
	**Non-severe (n=111, 59.68%)**	**Severe (n=75, 40.32%)**	
**Age (y/o), Median (IQR)**	42 (33 - 51)	66 (57 - 76)*	-
**Gender (n, %)**			
Male	45 (40.54)	45 (60.00)*	-
Female	66 (59.46)	30 (40.00)*	-
**Clinical categories (n, %)**			
Elevated blood pressure	6 (5.41)	10 (13.33)	-
Hypertension	15 (13.51)	20 (26.67)*	-
Diabetes	7 (6.31)	21 (28.00)*	-
Chronic lung diseases	7 (6.31)	10 (13.33)	-
Chronic kidney diseases	2 (1.8)	2 (2.67)	-
Gastrointestinal diseases	2 (1.8)	1 (1.33)	-
Malignant tumor	2 (1.8)	0 (0)	-
**Laboratory findings**			
CTnI (pg/mL)	5.68 (4.62 - 6.45)	7.00 (5.78 - 27.00)*	0 - 40.00
White blood cells ($\times 10^9$, cells/L)	5.07 (3.65 - 6.00)	5.98 (4.60 - 10.00)*	3.50 - 9.50
Neutrophils ($\times 10^9$, cells/L)	2.67 (2.15 - 3.92)	3.85 (3.02 - 8.27)*	1.80 - 6.30
Lymphocytes ($\times 10^9$, cells/L)	1.27 (0.96 - 1.73)	0.78 (0.48 - 1.29)*	1.10 - 3.20
Monocytes ($\times 10^9$, cells/L)	0.42 (0.34 - 0.54)	0.44 (0.29 - 0.69)	0.10 - 0.60
Procalcitonin (pg/mL)	36.00 (23.00 - 57.00)	67.50 (32.00 - 288.00)*	0 - 100.00
C-reactive protein (mg/L)	6.20 (0.50 - 28.00)	48.55 (7.40 - 81.50)*	0 - 10.00
Lactic dehydrogenase (U/L)	233.50 (187.50 - 292.00)	316.00 (235.00 - 454.00)*	120 - 250

Student’s *t* test, Mann-Whitney test, *χ2 test* and *Fisher's exact tests* were used to compare the age, gender, and clinical category between 2 groups. *P <0.05 is considered statistically significant.

Table 7. Univariate and multivariate analysis for clinical severity of COVID-19

	Odds ratio	95% CI	P
**Univariate analysis**			
Age (years)	1.12	1.09 - 1.15	<0.001*
Male (%)	2.20	1.21 - 4.00	0.010*
CTnI (pg/mL)	1.13	1.05 - 1.22	0.002*
White blood cells ($\times 10^9$, cells/L)	1.39	1.13 - 1.70	0.002*
Neutrophils ($\times 10^9$, cells/L)	1.50	1.19 - 1.90	0.001*
Lymphocytes ($\times 10^9$, cells/L)	0.23	0.10 - 0.54	0.001*
Procalcitonin (pg/mL)	1.01	1.00 - 1.01	0.069
C-reactive protein (mg/L)	1.02	1.01 - 1.04	0.002*
Lactic dehydrogenase (U/L)	1.01	1.00 - 1.01	0.001*
Hypertension (%)	2.489	1.185 - 5.226	0.016*
Diabetes (%)	5.78	2.31 - 14.45	<0.001*
**Multivariate analysis**			
Age (years)	1.11	1.07 - 1.16	<0.001*
Male (%)	1.38	0.57 - 3.37	0.479
CTnI (pg/mL)	1.08	1.01 - 1.15	0.018*
Hypertension (%)	7.19	2.55 - 20.31	<0.001*
Diabetes (%)	4.28	1.41 - 12.97	0.010*

The logistic regression model was used to determine factors associated with the clinical severity of COVID-19 according to Table 6, *P <0.05 is considered statistically significant.

Figure1

Click here to access/download;Figure;Fig1-ver26-small.tif

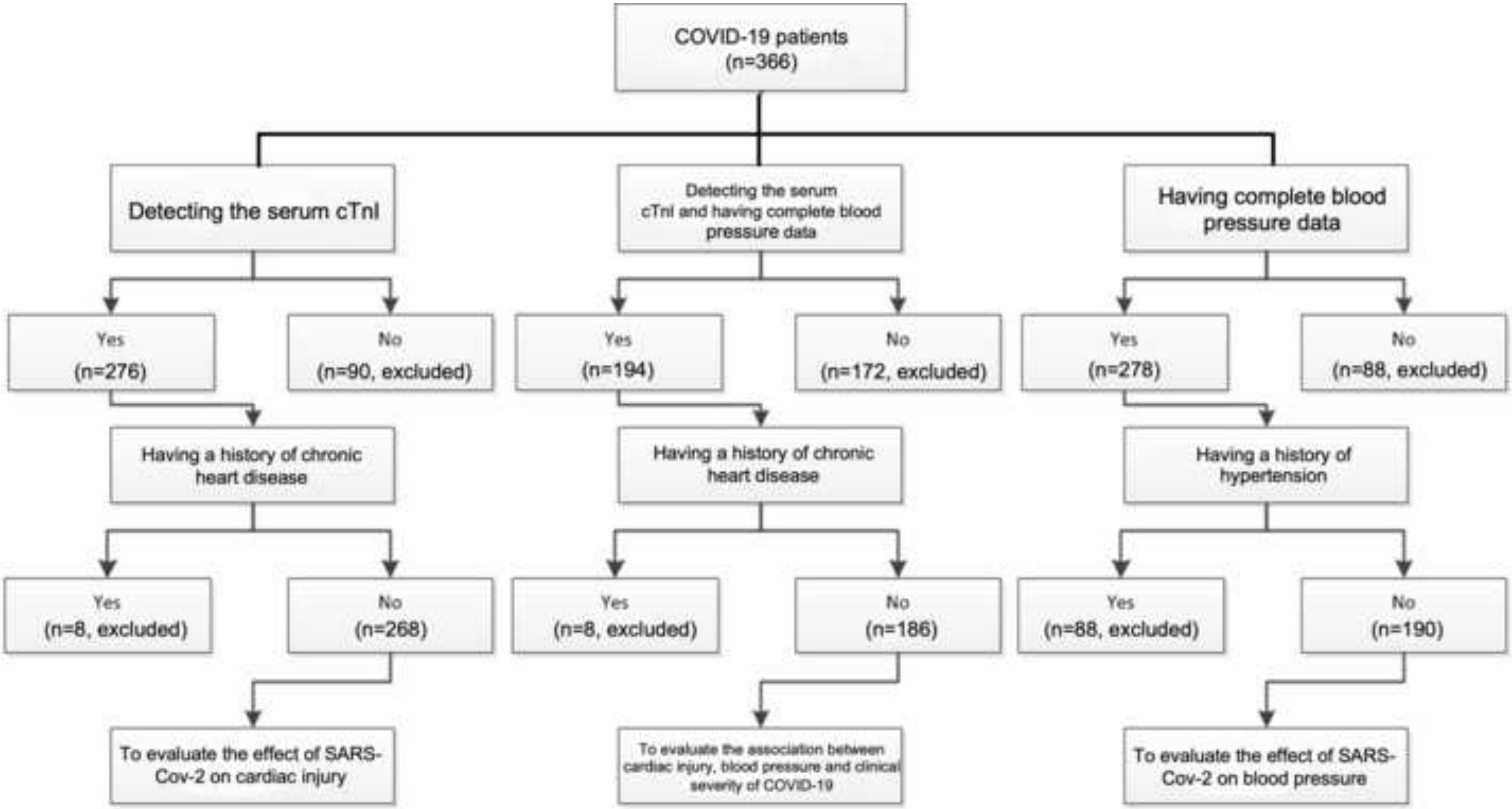

Figure2

Click here to access/download;Figure;Fig2-ver26-small.tif

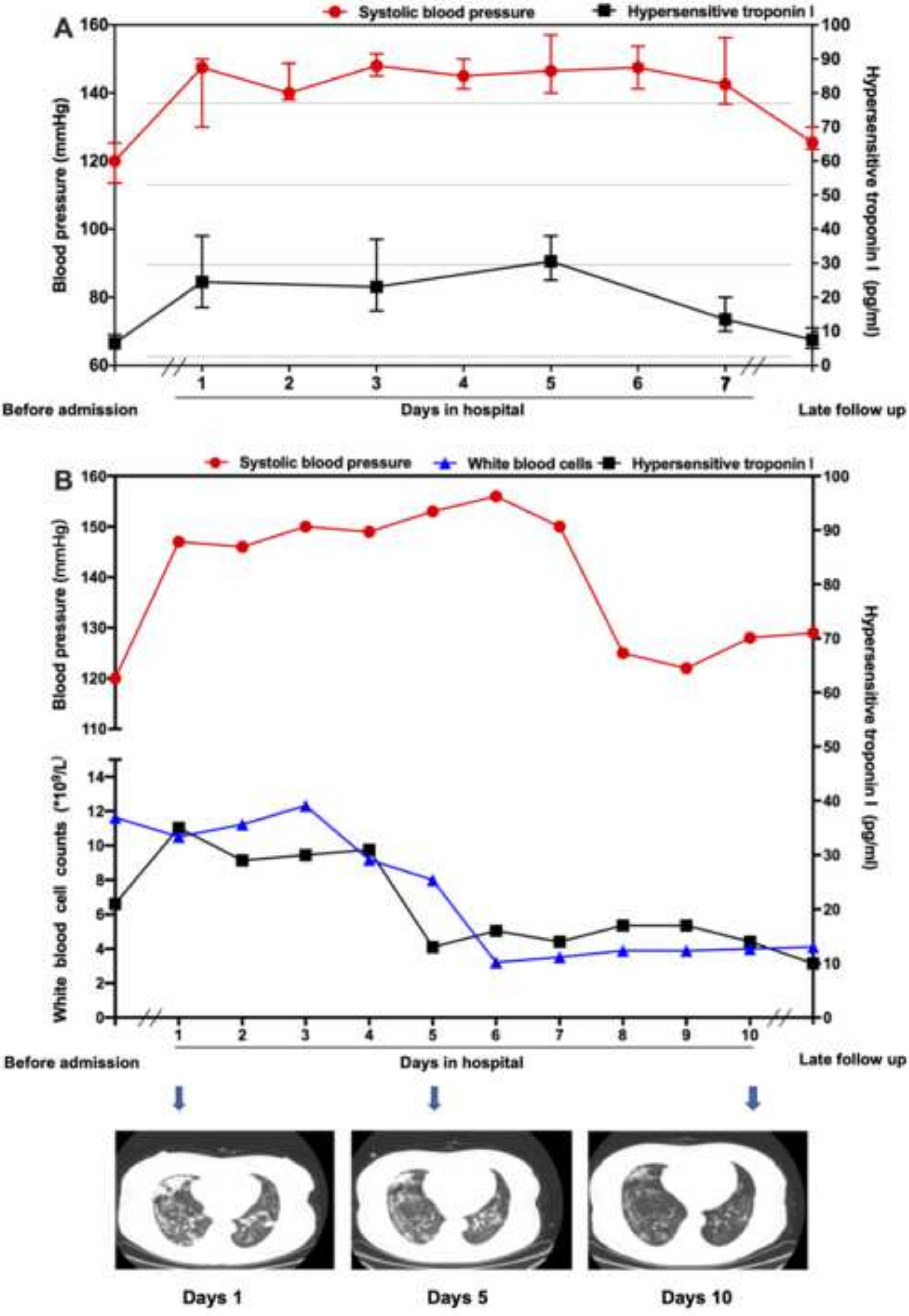

Figure3

Click here to access/download;Figure;Fig3-ver26-small.tif

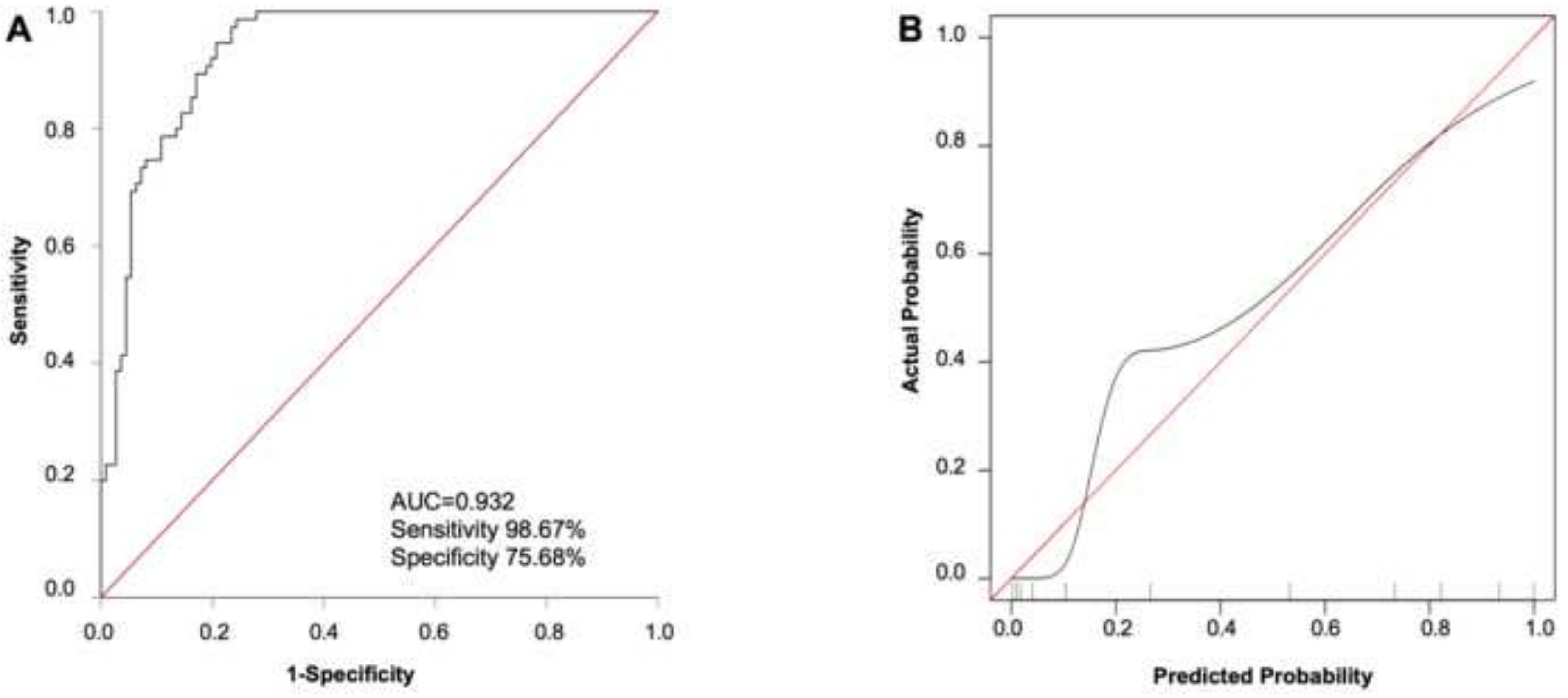

Supplement: Attachment — Submitted filename: PONE-D-21-02635_reviewer.pdf [file pone.0250815.s001.pdf]
